# Monitoring the Results of Foodborne Diseases in Sentinel Hospitals in Wenzhou City, China from 2014 to 2015

**Published:** 2018-05

**Authors:** Shi GUO, Dan LIN, Li-li WANG, Hong HU

**Affiliations:** 1. Dept. of Humanities and Management, Wannan Medical College, Wuhu, China; 2. Wenzhou Center for Disease Prevention and Control, Zhejiang, China

**Keywords:** Foodborne diseases, Sentinel hospital, Active monitoring

## Abstract

**Background:**

To investigate the epidemiologic distribution of foodborne diseases in Wenzhou City from 2014–2015, we analyzed the characteristics and main pathogens of foodborne disease outbreaks to provide a reference for the prevention, control, and early warning of foodborne diseases.

**Methods:**

A total of 9139 patients with foodborne diseases were included in the database of active monitoring from sentinel hospitals in Wenzhou City, China. *Salmonella*, *Shigella*, *Vibrio parahaemolyticus*, enteropathogenic *Escherichia coli,* and norovirus in the stool samples collected from patients with foodborne diseases were detected according to national standards.

**Results:**

There were 82 cases of *Salmonella*, 6 cases of *Shigella*, 393 cases of *V. parahaemolyticus*, 9 cases of diarrhoeagenic *E. coli*, and 143 cases of norovirus in the 9139 stool and anal swab samples, for a total detection rate of 6.98%. The peak season in which foodborne diseases occurred was from Jun–Oct. The high-risk populations were the 0–5, 16–25, 26–35, 35–45, and 46–55 yr age groups. Aquatic products accounted for the greatest proportion of suspected food, followed by meat, poultry, fruits, and vegetables.

**Conclusion:**

*V. parahaemolyticus* was the main pathogen of foodborne diseases in the Wenzhou urban area; *Salmonella* and norovirus were also foodborne disease pathogens. To prevent foodborne diseases, it is necessary to strengthen active monitoring, especially the sanitary management of seafood.

## Introduction

WHO defines foodborne diseases as “diseases causing the infection or poisoning of human bodies, usually caused by pathogens that enter bodies through ingestion.” Foodborne diseases are becoming a worldwide problem threatening the public health. Foodborne disease surveillance is an important part of the WHO global food security strategy. Five thousand people die of foodborne diseases each year in the United States (1). Bacterial foodborne disease is an important cause of morbidity and mortality. The foodborne disease active surveillance network performs routine surveillance for seven pathogenic bacteria (2); 12 bacterial pathogens are routinely surveilled in Australia (3), England (4), and other developed countries.

To carry out foodborne disease monitoring and establish a surveillance and report system of foodborne diseases is the basis of effective prevention and control of disease (5). On 24 Apr 2015, the People’s Republic of China passed the revised version of *The Law of Food Safety*, formally implemented on 1 Oct 2015. In the 2^nd^ chapter of the law, article 14 states “the country establishes a food safety risk monitoring system, to carry out the surveillance of foodborne diseases and food contamination.” Foodborne disease surveillance is still in the initial stage in China (6). Wenzhou City implemented foodborne disease surveillance at the end of 2011. Relying on sentinel hospitals and the Center For Disease Control and Prevention (CDC) in Wenzhou CDC, active and passive surveillance, and an early warning and control system for foodborne diseases are carried out to identify the spread of foodborne diseases, to determine the risk factors for foodborne diseases, and to formulate a prevention and control strategy for foodborne diseases, thus providing a scientific basis for health education of foodborne diseases and reducing risk (7).

Foodborne disease surveillance is usually faced with the problem that the data collected is not representative. To solve this problem, the international experience is called upon (8,9), and under the guidance of notice on the further strengthening of food safety monitoring, released by the National Health and Family Planning Commission, Wenzhou city and its 11 subordinate counties (cities and districts) carried out a comprehensive monitoring of foodborne diseases in 2014. Each county (city and district) selected a general hospital as the sentinel hospital for foodborne disease surveillance after unified training.

In the current study, all of the foodborne disease monitoring information in sentinel hospitals from 2014–2015 was analyzed, and the time, area, population distribution, and variation of food-borne pathogens were identified. The pathogens were analyzed to provide a correlation between the actual evidence from the epidemiologic investigation. Food contamination sources were analyzed to confirm the role in the outbreaks of foodborne diseases, improving early detection by the government, providing basic data, and the scientific basis for formulating prevention and control strategies.

## Materials and Methods

### Materials source

In this cross-sectional study, 12 sentinel hospitals were selected in Wenzhou City and actively monitored the medical records of foodborne diseases from 2014–2015. The patients were asked to complete the foodborne disease monitoring information table. Stool samples were collected and sent to the laboratory for isolation and cultivation. All participants gave informed consent and the study was approved by the local Ethics Committee.

### Case definition

The outpatient service cases involved patients with complaints of diarrheal symptoms with ≥ 3 bowel movements per day and abnormal stool characteristics (thin, watery, mucus or pus and blood), thus meeting the surveillance criteria. The patients were asked details if basic information, symptoms, 24 h dietary exposure history, and the stool or rectal swab samples were collected before the administration of drugs for pathogen detection.

### Testing items and methods

All medical specimens were collected and transported to the laboratory at room temperature in Cary-Blair within 24 h. The test items in each sentinel hospital included *Salmonella*, *Shigella*, *V. parahaemolyticus*, and diarrhoeagenic *E. coli*. Specimens from a sentinel hospital were chosen for norovirus detection. All inspections were carried out strictly according to the national standards (GB47894-2010, GB47895-2003, GB47897-2008, and GB47896-2003) using fluorescence quantitative polymerase chain reaction (PCR) and the testing procedures specified in *The Manual of 2014 National Foodborne Disease Monitoring Work*.

### Quality control and statistical analysis

Various forms of intensive training, standardized data collection, reporting, biological sample collection, and laboratory detection methods in all the sentinel hospitals were used to fully understand the purpose, content, methods, and significance of foodborne disease monitoring, improve the inspection rate of specimens, ensure complete, confirmed, and reliable data, and establish the stage-by-stage data review mechanism (10).

Analyses were performed using SPSS (ver. 12.0; SPSS, Inc., Chicago, IL, USA).

## Results

### Gender distribution of cases

A total of 9139 cases of foodborne diseases were monitored in 12 sentinel hospitals in Wenzhou from 2014–2015, of which 5074 male cases accounted for 55.5% of the total, and 4065 females accounted for the remaining 44.5%. The incidence of foodborne diseases between men and women was not statistically significant (*P*>0.05; Table 1).

**Table 1:** Gender distribution of foodborne disease cases in Wenzhou, 2014–2015

***Gender***	***2014***		***2015***		***Total***	
	***Number***	***Percent***	***Number***	***Percent***	***Number***	***Percent***
Male	2363	56	2711	55.1	5074	55.5
Female	1853	44	2212	44.9	4065	44.5
Total	4216	100.0	4923	100.0	9139	100.0

### Age distribution of cases

Based on the age distribution of cases from 2014–2015, the morbidity in the <5, 16–25, 26–35, and 36–45 yr age groups was higher, accounting for 69.3% of the total cases (Table 2).

**Table 2:** Age distribution of foodborne diseases cases in Wenzhou, 2014–2015

***Age group(yr)***	***2014***		***2015***		***Total***	
	***Number***	***Percent***	***Number***	***Percent***	***Number***	***Percent***
0-	1079	25.6	1329	27.0	2408	26.3
6-	217	5.1	249	5.1	466	5.1
16-	581	13.8	613	12.5	1194	13.1
26-	697	16.5	844	17.1	1541	16.8
36-	553	13.1	641	13.0	1194	13.1
46-	419	9.9	522	10.6	941	10.3
56-	333	7.9	370	7.5	703	7.7
66-	337	8.0	355	7.2	692	7.6
Total	4216	100.0	4923	100.0	9139	100

### Time distribution of cases

**Fig. 1:**
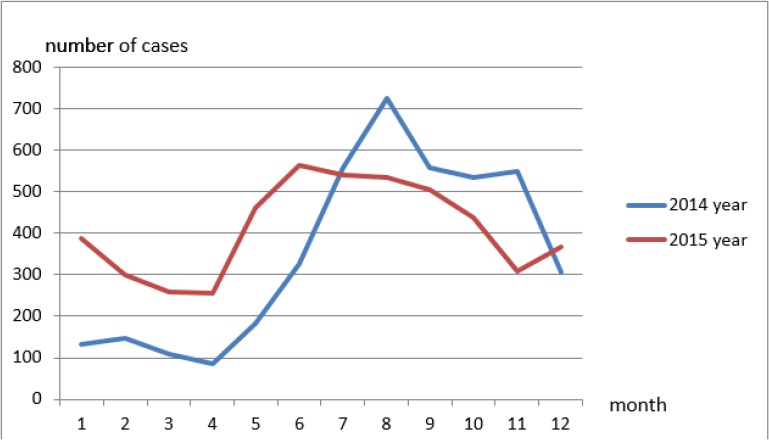
Time distribution of foodborne disease cases in Wenzhou, 2014–2015

Samples were collected from all patients suspected of foodborne diseases every month from 2014–2015. Based on the time distribution of cases in the 2-yr study period, foodborne diseases occurred every month. Morbidity was relatively low from Jan to Apr, with Apr being the lowest month of the year. Morbidity began to increase in May. In 2014, the peak occurred in Aug, which accounted for 17.20% of the annual cases, and the peak in 2015 occurred in Jun, which accounted for 11.50% of the annual cases. In the two years, the cases occurring in Jul to Nov were relatively concentrated (Fig. 1).

### Clinical manifestations

In 2014–2015, 9139 of the patients had diarrhea; 47.9% had abdominal pain, 24.7% had vomiting, 18.2% perceived weakness, and 17.7% had nausea. The clinical symptoms of patients from the 12 sentinel hospital patients are shown in Table 3.

**Table 3:** Clinical symptoms of foodborne diseases in Wenzhou city, 2014–2015

***Clinical symptoms***	***2014***		***2015***		***Total***	
	***Cases***	***Proportion***	***Cases***	***Proportion***	***Cases***	***Proportion***
Diarrhea	4216	100	4923	100.0	9139	100.0
Nausea	822	19.5	799	16.2	1621	17.7
Vomiting	1005	23.8	1249	25.4	2254	24.7
Weakness	982	23.2	678	13.8	1660	18.2
Abdominal pain	2107	50.0	2267	46.0	4374	47.9
Fever	398	9.4	382	7.8	780	8.5
Thirst	301	7.1	224	4.6	525	5.7
Vertigo	5	0.12	2	0.0	7	0.1
Chills	17	0.4	7	0.1	24	0.3
Dehydration	85	2.0	80	1.6	165	1.8
Pale complexion	169	1.5	47	1.0	216	2.4

### Pathogen detection

In 2014–2015, 9096 samples were collected from 12 sentinel hospitals. Six hundred thirty-five strains of pathogenic bacteria were detected and the detection rate was 6.98% (Table 4). Of the 635 strains of pathogenic bacteria, there were two strains of *V. vulnificus,* for a detection rate of 0.11%, 393 strains of *V. parahaemolyticus,* for a detection rate of 4.32%, 143 strains of norovirus, for a detection rate of 15.70%, 82 strains of *Salmonella,* for a detection rate of 0.90%, 6 strains of *Shigella,* for a detection rate of 0.07%, and 9 strains of diarrheagenic *E. coli,* for a detection rate 0.10%.

**Table 4:** Distribution of foodborne pathogens in Wenzhou city, 2014–2015

***Pathogens***	***2014***			***2015***			***Total***		

	Positive number	Number detected	Detection rate (%)	Positive number	Number detected	Detection rate (%)	Positive number	Number detected	Detection rate (%)
*Vibrio para-haemolyticus*	218	4190	5.20	175	4906	3.57	393	9096	4.32
*Salmonella*	44	4190	1.05	38	4906	0.77	82	9096	0.90
*Vibrio vulnificus*	2	1163	0.17	0	718	0.00	2	1881	0.11
diarrheogenic *Escherichia coli*	6	4190	0.14	3	4759	0.06	9	8949	0.10
*Shigella*	3	4190	0.07	3	4906	0.06	6	9096	0.07
Norovirus	48	395	12.15	95	516	18.41	143	911	15.70
Total	321	4190	7.66	314	4906	6.40	635	9096	6.98

### Exposure to suspicious food

**Fig. 2:**
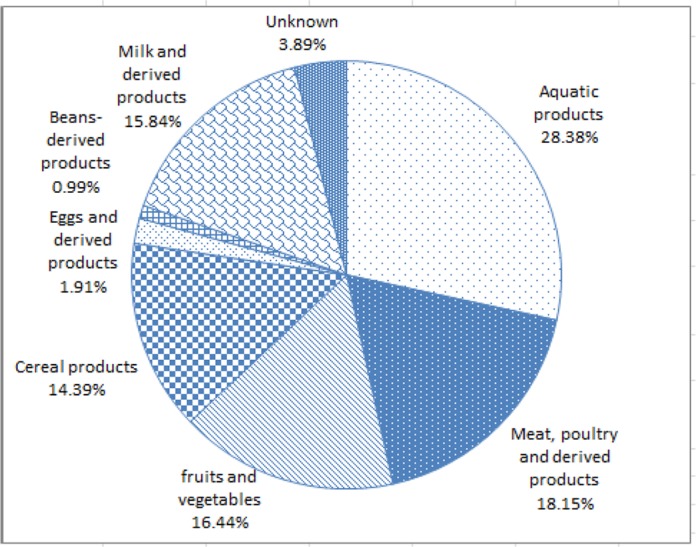
Exposure to suspicious food of foodborne diseases in Wenzhou, 2014–2015

Of all the suspicious foods that patients complained of, aquatic products accounted for 28.38%, which is the largest proportion, followed by meat, poultry, and their products accounting for 18.15%, fruits and vegetables accounting for 16.44%, and milk and dairy products accounting for 15.84%. All specific cases of suspicious food exposure are shown in Fig. 2.

## Discussion

Foodborne diseases, as one of the most extensive health problems in the world, are attracting more and more attention due to the harm to human health. A large number of dispersed foodborne diseases of short duration are easily ignored. This situation usually involves children, pregnant women, the elderly, and other people with impaired immunity. Foodborne diseases not only do harm to human health but also have a serious impact on the economic interests of the individual, family, society and the country, thus increasing the burden of disease. We selected 12 sentinel hospitals in Wenzhou city in Zhejiang Province to actively monitor the dispersed foodborne diseases and better understand the epidemiologic characteristics in the area.

### Time distribution

Bacterial foodborne diseases usually occur in summer due to the high temperature and humidity, which provides a good environment for the growth and production of bacteria. In summer, with decreased immunity, the human body produces less antibody synthesis. This leads to weakened resistance to various pathogenic bacteria, therefore enhancing the morbidity of foodborne diseases. According to the results of the current study, the number of hospital visits in Jun to Nov in the area was significantly higher than other months. This was in agreement with previous research results. Therefore, the prevention and control of summer season foodborne diseases, together with seasonal dietary sanitation, cannot be ignored.

### Distribution of the susceptible population

Children are a susceptible population of food-borne diseases. According to the report from the WHO, diarrhea is a foodborne disease. Every year, approximately 1.5 billion children <5 yr of age have diarrhea worldwide, with >2.5 million deaths (11), 70% caused by the biological contamination of food. In 2014–2015, the morbidity of foodborne diseases for the <5 yr age group is clearly higher than other age groups. Because infants of this age bracket are in the growth stage, impaired immunity, under-developed digestive function, and bad health habits all increase the dangers of pathogen infections. Therefore, infant-food hygiene safety is the focus of our attention. In addition to the high morbidity of infant infections, young adults from 16–45 yr of age also have high rates of infection, which may be in relation to the eating-places and health habits. This further confirms that in spite of active foodborne disease surveillance, food safety education and the knowledge of foodborne diseases should be popularized at the same time.

### Clinical symptoms

All 9139 cases had diarrhea, accompanied by abdominal pain, vomiting, nausea, and fatigue; a small number of patients had fevers, thirst, and other clinical symptoms.

### Distribution of exposure causes

Wenzhou is located in southeast Zhejiang Province; the southern border reaches the Oujiang River. The residents of Wenzhou have loved seafood since ancient times (12). Eating raw seafood is an important characteristic of the Wenzhou diet. From the data of exposure to suspicious food, foodborne diseases caused by aquatic products have a high morbidity. Meat and meat-derived products also lead to a relatively high morbidity. Thus, the region should strengthen the management of aquatic products. Aquatic products should be cooked thoroughly and raw seafood should not be eaten to prevent the cross-contamination of tableware, other foods, and seafood. Foodborne diseases caused by vegetables, fruits, and dairy products are also implicated. In spite of the cross-contamination between seafood, some residents eat overnight leftovers, which is one of the causes that trigger foodborne diseases (13). The children’s foodborne diseases are mainly caused by improper drinking of exposed dairy products. All of these habits suggest that we should strengthen awareness of food safety and guard against foodborne diseases caused by food spoilage.

### Pathogen distribution

Norovirus, which is one of the most serious foodborne disease pathogens, is the main pathogen that causes worldwide epidemic acute non-bacterial gastroenteritis (14). Approximately 90% of non-bacterial diarrheas are caused by the virus. The objects of norovirus infection can be people of all age groups, of which the elderly, children, and immunodeficient are at high risk (15). In 2014, Wenzhou city chose a sentinel hospital to collect specimens for norovirus detection. In 2014–2015, the detection rate of norovirus was 15.70%, which is much higher than the detection rate of other pathogenic bacteria. This indicates that the sporadic characteristic of the foodborne diseases caused by norovirus in Wenzhou City.

*V. parahaemolyticus* is a Gram-negative bacterium, which exists in the coastal waters, mainly, polluting the marine products (16,17). Local dietary habits of seafood are the main cause of infection of *V. parahaemolyticus*. In 12 sentinel hospitals in Wenzhou City, *V. parahaemolyticus* is also the main pathogen detected, with a detection rate of 4.32%. The result is inconsistent with the results of another study. Specifically, detection of *V. parahaemolyticus* in Jiangsu Province and Shanghai city (China) was also high (18). Only through the analysis of a large volume of data, can the various rules and tendency of foodborne diseases be determined. Therefore, better health education for community residents will be provided to achieve the goal of preventing diseases and protecting human health (19).

## Conclusion

*V. parahaemolyticus* is the main pathogen of food-borne diseases in the Wenzhou urban area and the risk of *Salmonella* and norovirus also exist. To prevent foodborne diseases, it is necessary to strengthen active monitoring, especially the sanitary management of seafood. According to the results of 2 yr of active monitoring of foodborne diseases in Wenzhou, the information and data reflect the condition of foodborne diseases in the city. To effectively monitor and objectively release the early warning of food safety risk, an effective long-term monitoring system is needed.

## Ethical Considerations

All ethical issues, including plagiarism, informed consent, misconduct, data fabrication and/or falsification, double publication and/or submission, and redundancy, have been completely observed by the author.
